# Phenotypic and Genotypic Analysis of Antimicrobial Resistance in *Mycoplasma hyopneumoniae* Isolated from Pigs with Enzootic Pneumonia in Australia

**DOI:** 10.3390/pathogens13121044

**Published:** 2024-11-28

**Authors:** Raziallah Jafari Jozani, Mauida F. Hasoon Al Khallawi, Hanh Thi Hong Nguyen, Majed H. Mohammed, Kiro Petrovski, Yan Ren, Darren Trott, Farhid Hemmatzadeh, Wai Yee Low

**Affiliations:** 1 The Australian Centre for Antimicrobial Resistance Ecology, University of Adelaide, Adelaide, SA 5005, Australia; mauida.alkhallawi@adelaide.edu.au (M.F.H.A.K.); nguyenhanhniah@gmail.com (H.T.H.N.); kiro.petrovski@adelaide.edu.au (K.P.); darren.trott@adelaide.edu.au (D.T.); farhid.hemmatzadeh@adelaide.edu.au (F.H.); 2 The Davies Livestock Research Centre, School of Animal and Veterinary Sciences, University of Adelaide, Adelaide, Adelaide, SA 5005, Australia; kelly.ren@adelaide.edu.au

**Keywords:** *Mycoplasma hyopneumoniae*, minimum inhibitory concentration (MIC), pigs, enzootic pneumonia, whole-genome sequencing

## Abstract

*Mycoplasma hyopneumoniae*, an important cause of enzootic pneumonia in pigs in many countries, has recently been shown to exhibit reduced susceptibility to several antimicrobial classes. In the present study, a total of 185 pig lung tissue samples were collected from abattoirs in Australia, from which 21 isolates of *M. hyopneumoniae* were obtained. The antimicrobial resistance profile of the isolates was determined for 12 antimicrobials using minimum inhibitory concentration (MIC) testing, and a subset (*n* = 14) underwent whole-genome sequence analysis. MIC testing revealed uniformly low values for enrofloxacin (≤1 μg/mL), florfenicol (≤8 μg/mL), lincomycin (≤4 μg/mL), spectinomycin (≤4 μg/mL), tetracycline (≤0.5 μg/mL), tiamulin (≤2 μg/mL), tildipirosin (≤4 μg/mL), tilmicosin (≤16 μg/mL) tulathromycin (≤2 μg/mL), and tylosin (≤2 μg/mL). Higher MICs were observed for erythromycin (MIC range: 16–32 μg/mL), gamithromycin, and tilmicosin (MIC range of both: 32–64 μg/mL). Whole-genome sequencing of the isolates and additional screening using mismatch amplification mutation assay PCR did not identify any known genetic resistance markers within 23S rRNA (macrolides), DNA gyrase A, and topoisomerase IV genes (fluoroquinolones). The WGS data also indicated that the Australian *M. hyopneumoniae* isolates exhibited limited genetic diversity and formed a distinct monophylectic clade when compared to isolates from other countries. These findings indicate that Australian *M. hyopneumoniae* likely remains susceptible to the major antimicrobials used to treat enzootic pneumonia in pigs and have evolved in isolation from strains identified in other pig-producing countries.

## 1. Introduction

Bacterial respiratory diseases in swine are responsible for great economic loss and are often initiated by *Mycoplasma hyopneumoniae*. *M. hyopneumoniae* is considered the principal etiological agent of enzootic pneumonia (EP), a condition responsible for chronic respiratory signs and high morbidity in grower and finisher pigs [1]. Both therapeutic use and prophylactic use of antimicrobials are important in the management of EP [2,3,4]. However, there have been several reports of antimicrobial resistance development among *M. hyopneumoniae* isolates, which complicates strategies for the treatment of EP and demand a better understanding of resistance profiles and mechanisms [5,6,7].

The challenge in managing antimicrobial resistance development in *M. hyopneumoniae* is compounded further by a significant gap in the literature correlating whole-genome sequencing (WGS)-based genetic profiles with phenotypic antimicrobial susceptibility data [8]. This gap primarily results from the fastidious nature of *M. hyopneumoniae*, making the isolation process lengthy and costly, and hence, the successful isolation of the pathogen from clinical samples is rare. This difficulty is clearly reflected in the relatively low number of whole-genome sequences of this species available in GenBank in comparison to other important bacterial pathogens of animals [9,10]. Hence, little is known about the genetic markers associated with AMR and the mechanisms driving resistance in *M. hyopneumoniae*, an understanding of which is instrumental for disease management and treatment strategies. Identification of specific markers (usually point mutations) in the *Mycoplasma* genome that impart resistance can help in the design of improved molecular-based diagnostic tools for a more targeted approach to identify resistant strains [11,12]. Furthermore, understanding the mechanisms underlying AMR can provide critical insights for predicting future resistance trends and updating guidelines to control and prevent EP [13].

Studies on the antimicrobial resistance of *M. hyopneumoniae* strains are currently insufficient and should adopt a proactive rather than reactive approach [4,14]. Although the need for such studies is acute, there are still many unanswered questions on the exact nature of the genetic determinants of AMR and the impact these determinants have upon the susceptibility of the pathogen to the various antimicrobials [6,7,11,15]. According to previous studies, mutations in the genes encoding the topoisomerase enzymes, *gyrA* and *parC*, were found to correlate with fluoroquinolone resistance, while mutations in the 23S rRNA gene correlate with resistance against macrolides [9,13]. Although there is no study specifically tracking the evolution of AMR in a particular geographical location, Gautier-Bouchardon [16] compared the minimum inhibitory concentration (MIC) profiles of *M. hyopneumoniae* from various locations in Europe across time (pre and post the year 2000) and found that more recent isolates had higher MIC ranges for tetracyclines, macrolides, and fluoroquinolones. In contrast, the MIC values for aminoglycosides were lower for isolates collected after 2000 [11]. These reports show how the MIC profile of *M. hyopneumoniae* changes over time. Due to the lack of detailed analyses that integrate antimicrobial susceptibility profiles with whole-genome sequence data obtained for *M. hyopneumoniae* isolates from different geographical regions [8,17], the present study analyzed the antimicrobial susceptibility and genomic characteristics of Australian isolates of *M. hyopneumoniae*, the latter in comparison to publicly available sequences. MIC profile data for the isolates were generated, and whole-genome sequencing was carried out in order to identify possible genetic markers associated with AMR. Furthermore, a mismatch amplification mutation assay (MAMA) based on polymerase chain reaction (PCR) chemistry was employed to validate the sequencing results by focusing on previously reported genetic mutations in *M. hyopneumoniae* associated with antimicrobial resistance.

## 2. Materials and Methods

### 2.1. Study Design and Sample Collection

A total of 185 lung tissue samples from pigs with post-mortem findings of EP such as consolidation of the lung tissue with a red-to-grey color change were collected from abattoirs across Australia and submitted to the PC2 laboratory at the University of Adelaide Roseworthy campus for molecular detection and *Mycoplasma* culture. Approximately, samples of 20 to 40 g of affected lung tissues were aseptically collected in 50 mL sterile plastic tubes containing *M. hyopneumoniae*-specific media, namely, Friis broth media. The tubes were transported in cool boxes to the laboratory within 24 to 48 h of collection.

### 2.2. Molecular Detection of M. hyopneumoniae and Other Porcine Mycoplasmas

Upon receipt in the laboratory, lung tissue samples were subjected to DNA extraction using the DNeasy Blood & Tissue Kit (QIAGEN, Hilden Germany) according to the manufacturer’s instructions. The polymerase chain reaction (PCR) assay was then applied to the extracted DNA, utilizing primers specific to the 16S–23S rRNA intergenic spacer region [18]. Amplicon size allowed for the differentiation of three porcine *Mycoplasma* species: *M. hyopneumoniae*, *Mycoplasma hyorhinis*, and *Mycoplasma flocculare* (Table 1). An additional *M. hyopneumoniae*-specific PCR test targeting a segment of the 16S rRNA gene was also included, given that the expected sizes of the products for *M. hyopneumoniae* and *M. flocculare* are similar (Table 1). PCR tests utilized Premix Taq (MyTaq™ Red DNA Polymerase, Bioline, Memphis, TN, USA) under the following thermal conditions: 30 cycles of denaturation at 95 °C for 30 s, annealing at 55 °C (16S–23S rRNA intergenic spacer region) or 56 °C (16S rRNA) for 90 s, and elongation at 72 °C for 1 min. Afterward, a final elongation step was performed at 72 °C for 10 min, resulting in the desired PCR product. Electrophoresis of PCR products was performed on a 10 cm agarose gel at 100 V for 30 min. DNA from *M. hyopneumoniae* type strain J (ATCC 25934) and *E. coli* (ATCC 25922) were considered as positive and negative controls, respectively. Molecular-biology-grade water (Thermo Fisher Scientific, Adelaide, Australia) was also used as a technical negative control. Visualization of PCR products was conducted straight after thermal cycling using a gel documentation system (Gel Doc XR+, Bio-Rad, Hercules, CA, USA).

### 2.3. Bacterial Isolation and DNA Extraction

Samples that tested positive for *M. hyopneumoniae* by PCR were subjected to *Mycoplasma* culture and isolation in Friis broth and on Friis agar to obtain isolates. Friis broth and agar were formulated following published protocols [19]. The same batch of pig (Gibco Batch 2254787) and horse (Gibco Batch 2540511) sera were used in all experiments, and each was heat-inactivated at 56˚ C for 20 min. Phenol red at 0.6% was used as a pH color-sensitive indicator of microbial growth, and the pH was adjusted to 7.4 with 1.0M HCl. Friis agar plates were prepared by mixing 1.8% agar No.1 (Thermo Fisher Scientific, Australia) and 0.01% (*w*/*v*) diethylaminomethyl dextran (Sigma-Alderich, US) with Friis broth base at 55˚C, as proposed by Cook et al. [19]. The broth cultures were incubated in microaerophilic jars containing a microaerophilic sachet (Thermo Fisher Scientific, Australia) and incubated at 37 °C until a color change to orange/yellow was observed. The identity of the isolates was confirmed using the same PCR methods as described above. To obtain pure isolates, a single colony from the Friis agar was subcultured into Friis broth and incubated. This process was repeated through three rounds of subculturing from Friis agar to Friis broth and back to Friis agar. Once a pure isolate was confirmed through PCR, it was cultured in Friis broth, and the bacterial pellet was collected by centrifugation at 18,000 rpm for 40 min at 4 °C. DNA was extracted from bacterial pellets obtained from cultured isolates using a DNeasy Blood & Tissue Kit (QIAGEN, Germany) according to the manufacturer’s instructions. Briefly, bacterial pellets were lysed using the kit’s lysis buffer, followed by DNA extraction using silica binding columns. The DNA eluted using Tris-EDTA buffer and the quantity of the extracted DNA was measured using a nanophotometer (NanoDrop One^C^ Microvolume UV-Vis, ThermoFisher, Waltham, MA, USA).

### 2.4. Antimicrobial Susceptibility Testing

MIC testing was carried out using the broth microdilution method of Hannan [20] with some modifications. The *M. hyopneumoniae* reference strain ATCC 25934 was used as a susceptible quality control reference strain in each MIC testing run. All isolates derived from pure cultures were preserved at −80 °C in liquid Friis broth–glycerol (15% *v*/*v*) medium. The following antimicrobials were purchased from Sigma-Aldrich Co. (Burlington, MA, USA): enrofloxacin (ENO), erythromycin (ERY), florfenicol (FFL), gamithromycin (GAM), lincomycin (LCM), spectinomycin (SPT), tetracycline (TET), tiamulin (TIM), tildipirosin (TIP), tilmicosin (TIL), tulathromycin (TUL), and tylosin (TYL). Each compound was stored as per the supplier’s instructions. The antimicrobial stock solutions were prepared following the CLSI guidelines [21]. Briefly, each antimicrobial compound was weighed (0.2560 g) and dissolved in deionized water to make a 50 mL stock solution with a final concentration of 5120 µg/mL. Stock solutions were aliquoted into 5 mL sterile tubes, labelled, and stored at −80 °C. For MIC determinations, stock solutions were thawed, and further working dilutions were prepared in Friis medium following the CLSI guidelines (CSLI, 2018). Each *M. hyopneumoniae* isolate was subcultured from agar or frozen stocks into 10 mL Friis broth medium and incubated at 37 °C until a typical color change was observed, and the required CFU/mL was reached after 3–15 days of incubation. The broth cultures were then divided into 1 mL aliquots and frozen at −80 °C. To determine cell density, aliquots were thawed, serially diluted to 10^−5^ in Friis broth medium, and applied to solidified Friis medium using an L-shaped sterile plastic spreader. Plates were incubated at 37 °C in air + 5% CO_2_ for 3–15 days, and colonies were enumerated under a stereomicroscope (Leica-M80, Germany) (125× magnification) to calculate CFU per mL.

Sterile polystyrene microtiter plates (Corning^®^, Corning, NY, USA) were used, with each well containing 100 µL of the diluted solutions. A positive control (bacteria+media) and negative control (media only) were included. Frozen broth cultures were thawed and incubated at 37 °C for 1 h. Cultures were then diluted in Friis broth medium to a theoretical density of 1 × 10^6^ CFU/mL and used to inoculate the microtiter wells. The final range of concentrations tested was 0.25–128 µg/mL. Inoculum density was confirmed by viable counts from positive control wells. Microtiter plates were incubated at 37° C for 3 to 5 days or until a color change/point pellet was observed. MIC was determined as the lowest concentration that completely inhibited growth. Plates were incubated for an additional 48 h if necessary to obtain a clear MIC determination.

### 2.5. Mismatch Amplification Mutation Assay (MAMA) PCR for AMR Marker Detection

To enhance our understanding of AMR, we developed 5 PCR tests targeting known resistance genes and mutations in the 23S rRNA, DNA gyrase A, and topoisomerase IV genes. Mismatched PCR was used to detect AMR markers in *M. hyopneumoniae* isolates. These PCR tests were developed based on a previously published paper [22] with some modifications. Primers specific to AMR genes were used, and PCR reactions were set up similarly to the detection PCR with specific annealing temperatures and product sizes, as listed in Table 2.

### 2.6. Whole-Genome Sequencing

Whole-genome sequencing (WGS) analysis was conducted on 14 *M. hyopneumoniae* isolates that successfully passed quality control steps to identify genetic markers associated with antimicrobial resistance (AMR). DNA quality was assessed using an Agilent Tape Station 2200 (Agilent Technologies, Santa Clara, CA, USA). Whole-genome sequencing (WGS) was performed on the NextSeq 550 platform using a NextSeq MID-output (2 × 150 bp) paired-end sequencing kit. Libraries were prepared with a Nextera XT Library preparation kit. Reads were trimmed with Trimmomatic v0.38 [23], and quality was checked with FASTQC v0.11.4 [24]. Genome assemblies were performed using SPAdes v3.12.0 [25], and assembly quality was checked with Quast v4.5. Antimicrobial resistance genes (ARGs) were predicted using the Antibiotic Resistance Genes Database (ARDB), Comprehensive Antibiotic Resistance Database (CARD), PointFinder, and ResFinder databases.

Publicly available WGS data for *M. hyopneumoniae* were downloaded from the NCBI. The genetic relationships between our isolates in Australia and public isolates were examined using single-nucleotide polymorphisms (SNPs) found from mapping WGS reads to a *M. hyopneumoniae* complete genome (NCBI Assembly Accession: NC_021283.1). The software Snippy v4.6.0 (https://github.com/tseemann/snippy (accessed on 02 June 2024)) was used to call common SNPs in all isolates. Using these SNPs, a maximum likelihood (ML) tree was constructed with RAxML v8.2.10 [26] using the model GTRCAT, and a rapid bootstrap analysis with 100 bootstraps for the best-scoring ML tree was carried out. This was followed by recombination removal using ClonalFrameML v1.12 [27]. The final phylogenetic tree was manipulated with iTOL (https://itol.embl.de/ (accessed on 02 June 2024)) for display.

### 2.7. Statistical Analysis

The MIC_50_, MIC_90_, MIC range, median, mode, and geometric mean MIC were determined for each antimicrobial tested. Data were analyzed using the Microsoft Excel software.

## 3. Results

### 3.1. Species-Specific PCR Results, and Isolation and Identification of Mycoplasma Species

**Figure 1:**
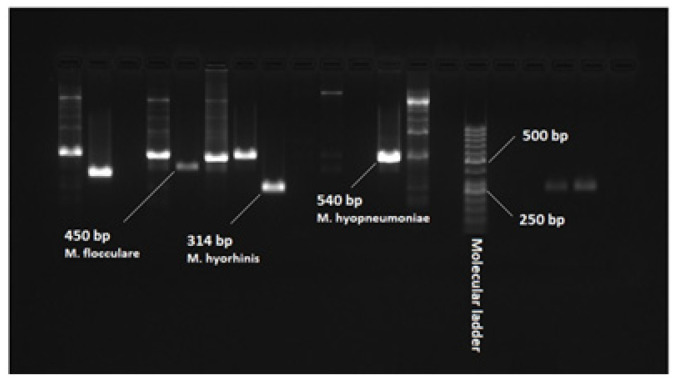
PCR results from the pig lung samples for *Mycoplasma* testing using primers targeting intergenic spacer (IGS) region between 16S and 23S rRNA genes (species-specific PCR). *M. hyopneumoniae* produced a 540 bp product, *M. flocculare* a 450 bp product, and *M. hyorhinis* a 314 bp product.

**Figure 2:**
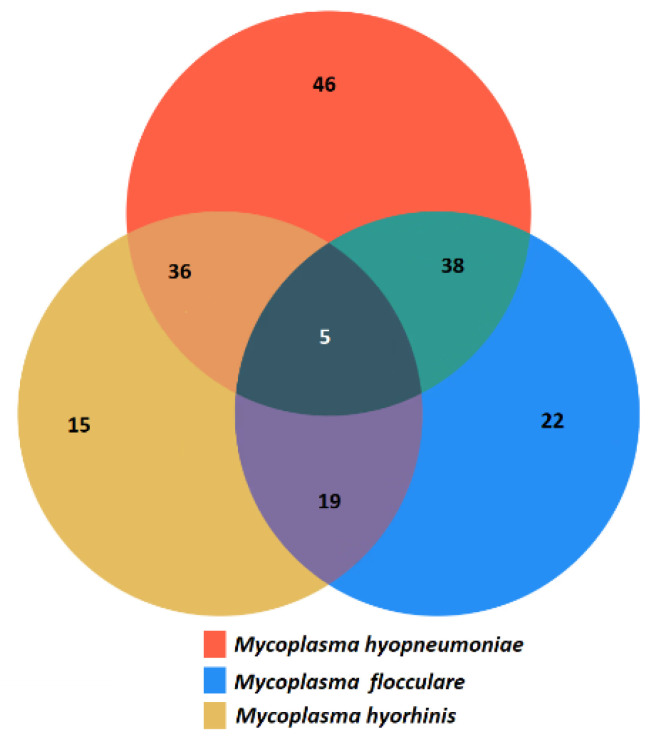
Distribution of *Mycoplasma* species in pig lung samples by specific intergenic spacer region PCR.

Figure 1 shows the PCR results targeting the 16S rRNA gene of *M. hyopneumoniae*, with positive samples producing a 645 bp product. The PCR tests were performed using intergenic spacer primers (IGS) to identify *M. hyopneumoniae* (540 bp product), *M. flocculare* (450 bp product), and *M. hyorhinis* (314 bp product). In total, 181/185 (97.8%) of the lung samples obtained from pigs diagnosed with EP at slaughter were positive by PCR (Figure 1) for at least one species of *Mycoplasma*, and 125/185 (69.1%) were positive by PCR for *M. hyopneumoniae*. Additionally, 74/125 (59.2%) of the *M. hyopneumoniae* PCR-positive lung tissue samples were also PCR-positive for either *M. hyorhinis* or *M. flocculare*. Co-infections with multiple species were commonly detected (Figure 2).

**Figure 3:**
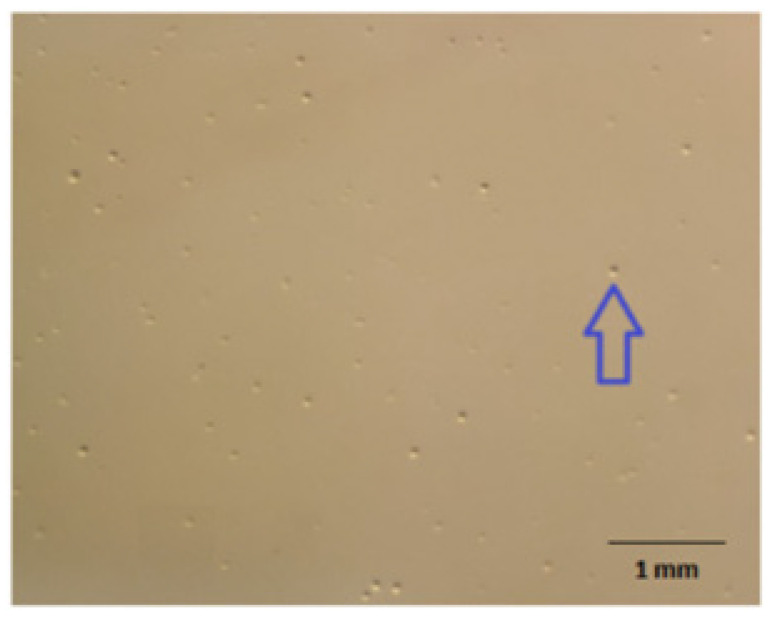
A pure culture of *M. hyopneumoniae* growing on Friis agar visualized under a stereomicroscope at 120× magnification. After the third subculture, the colonies became significantly larger, reaching 5–10 µM in diameter. A bigger colony is shown by the arrow.

**Figure 4:**
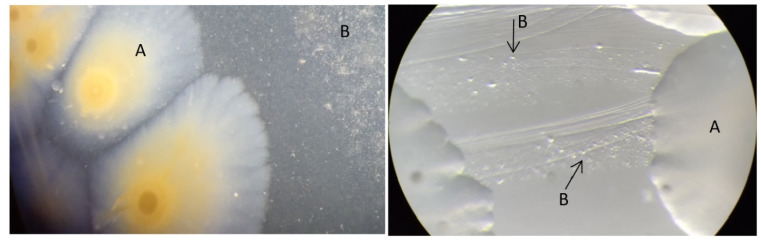
*M. hyorhinis* colonies (A) and *M. hyopneumoniae* colonies (B) growing on solid Friss agar. *M. hyorhinis* colonies had a typical fried-egg appearance and were 1–2 mm in diameter, whereas *M. hyopneumoniae* colonies were very small, only 10–20 µM in size.

Initial attempts to culture the 125 PCR-positive lung tissue samples for *M. hyopneumoniae* were hindered by a lack of growth or extremely slow growth. Only a small proportion of the *M. hyopneumoniae* PCR-positive lung samples (21/125; 16.8%) eventually yielded pure cultures of the pathogen after three rounds of cloning and subculturing (Figure 3), which were confirmed to be *M. hyopneumoniae* by PCR and/or whole-genome sequencing. The appearance of *M. hyorhinis* and *M. hyopneumoniae* in the mixed culture combined with the fast-growing nature and larger size of the *M. hyorhinis* fried-egg-shaped colonies (Figure 4) also significantly impeded efforts to isolate *M. hyopneumoniae* in a pure culture. Of the 21 *M. hyopneumoniae* isolates obtained, 14 were from Victoria, 3 were from New South Wales, 2 were from Queensland, and 2 were from South Australia.

### 3.2. Antimicrobial Susceptibility Testing

The MIC testing results for the Australian *M. hyopneumoniae* isolates revealed generally low MICs for the majority of the tested antimicrobials (Table 3 and Table 4). However, higher MICs were observed for erythromycin (MIC range = 32–64 μg/mL), gamithromycin, and tilmicosin (MIC range for both = 16–32 μg/mL).

### 3.3. Whole-Genome Sequencing

Sequences were screened for the presence of known AMR genes as well as possible mutations in key target genes associated with antimicrobial resistance against the ARDB, CARD, PointFinder, and ResFinder databases. No AMR genes were detected in any of the isolates. No specific genetic markers in the 23S rRNA gene were identified that could be associated with the high erythromycin, gamithromycin, and tilmicosin MICs.

### 3.4. Phylogenetic Results

**Figure 5:**
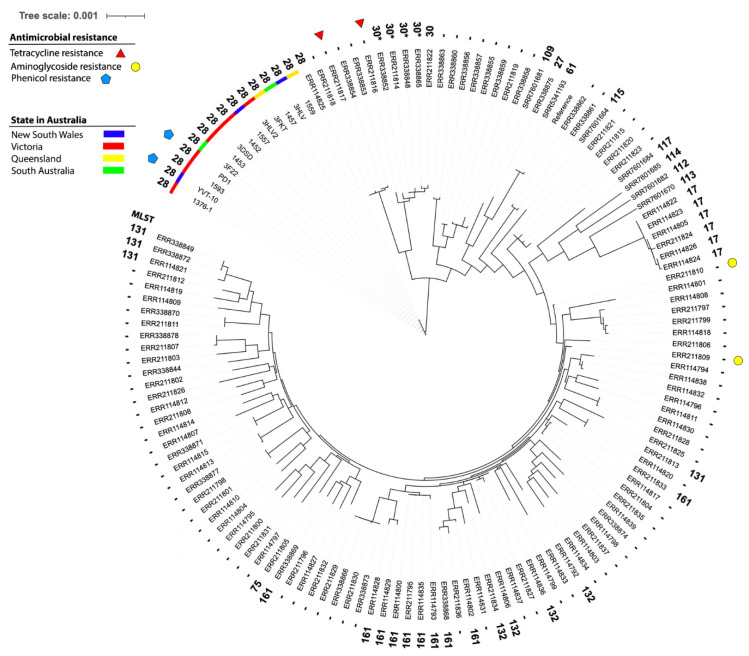
Phylogenetic tree of all available *M. hyopneumoniae* isolates in NCBI compared to the 14 Australian sequenced isolates in this study. Australian *M. hyopneumoniae* isolates formed an outlier with low levels of diversity (SNPs).

Overall, there were 15,540 common SNPs identified by snippy for the phylogenetic analysis. The result shows a short genetic distance among the 14 Australian isolates from states including New South Wales, Victoria, Queensland, and South Australia (Figure 5). The raw WGS data of those isolates are available in the SRA under BioProject PRJNA1117611. The results of the collected *M. hyopneumoniae* isolates according to MLST and antimicrobial resistance are marked on the tree.

### 3.5. Mismatched PCR for AMR Marker Detection

Mismatched PCR tests aimed to detect both wild-type and resistant isolates by incorporating mismatched primers specific to conserved and mutated sequences.

No resistance genes were detected in the 23S rRNA and DNA gyrase A genes among the tested isolates, indicating a lack of genetic basis for resistance to macrolides and fluoroquinolones. Similarly, PCR screening for mutations in topoisomerase IV genes showed no evidence of quinolone resistance.

Florfenicol showed relatively low MICs (≤8 μg/mL) in the present study; however, two *M. hyopneumoniae* isolates (from VIC and NSW) showed a likelihood for a genotypic profile of phenicol resistance (Figure 5).

## 4. Discussion

This study investigated the AMR profiles and genomic characteristics of *M. hyopneumoniae* isolated from Australian pigs suffering from EP. Overall, the MIC profiling showed uniformly low MICs for the majority of the tested antibiotics, with higher MICs (16–64 μg/mL) only reported for macrolides like erythromycin, gamithromycin, and tilmicosin. No known genetic markers for resistance that could explain these high macrolide MICs were identified from the WGS analysis. Furthermore, WGS identified an overall low genetic diversity among the Australian isolates, which formed a single monophyletic clade. Such findings are suggestive of the observed higher MIC values to macrolides at a phenotypic rather than genotypic level and point out that more research is needed to investigate the possible mechanism behind these findings.

Previous research has shown a range of high MIC values among different *Mycoplasma* species, likely influenced by the use of various antimicrobial agents. These values in many *Mycoplasma* species show the urgent need for standardized susceptibility testing methods, which are essential for harmonizing antimicrobial testing and determining breakpoints specific to *Mycoplasma* pathogens [16,28,29,30]. *Mycoplasma hyopneumoniae* is generally susceptible to macrolides, tetracyclines, and fluoroquinolones, although higher MIC values for macrolides and tetracyclines are more frequently observed, while most strains remain susceptible to fluoroquinolones [11,16].

Considering the lack of fluoroquinolone use in food-producing animals in Australia [31], the consistently low MICs for most antimicrobials, including fluoroquinolones [8], are remarkable. This aligns with global trends, where fluoroquinolone susceptibility in *M. hyopneumoniae* remains relatively high, particularly in regions with controlled use. In Australia, this is the result of strict regulations on the use of fluoroquinolones in food animals, which help prevent the selection of resistant strains [16,31]. Our results are in line with earlier studies that reported similar susceptibility profiles for *M. hyopneumoniae* isolates in other regions (e.g., Europe), where fluoroquinolones, tetracyclines, and pleuromutilins also appeared effective *in vitro* against *M. hyopneumoniae* [8].

Studies such as those by Thacker and Minion [32] and Felde et al. [8] support this finding, showing that the frequency of high MIC values for fluoroquinolone in *M. hyopneumoniae* remains low in populations that do not undergo extensive fluoroquinolone treatment. In Europe, where fluoroquinolone usage is more prevalent, higher MIC values are reported more frequently [13], but even there, the majority of isolates remain susceptible [16].

Nonetheless, a study by Vicca et al. [6] pointed out that fluoroquinolone resistance can rapidly emerge in *M. hyopneumoniae* under selective pressure, as seen in other regions. Conversely, Le Carrou et al. [33] showed that low MIC values persist when fluoroquinolone use is limited, as seen in Australia. The effectiveness of tylvalosin in controlling porcine enzootic pneumonia (EP) indicates the importance of selecting appropriate antimicrobials based on susceptibility profiles [34]. This emphasizes the importance of ongoing surveillance to monitor any shifts in antimicrobial susceptibility patterns, especially as antimicrobial use policies evolve over time.

The absence of genetic markers for decreased susceptibility to macrolides in our *M. hyopneumoniae* isolates contrasts with other studies that have identified such markers in different *Mycoplasma* species [13]. Our elevated MICs for some macrolides such as erythromycin, gamithromycin, and tilmicosin, despite the absence of 23S rRNA mutations, indicate potential non-genetic resistance mechanisms. Monitoring the antimicrobial susceptibility of *M. hyopneumoniae* in Europe showed high MIC values despite the absence of corresponding genetic markers [35]. In contrast, Sulyok et al. [13] identified 23S rRNA mutations as a primary mechanism of resistance in another European-oriented study, suggesting geographic variation in resistance mechanisms. This suggests that resistance mechanisms may vary by region and are shaped by local antimicrobial usage practices. An investigation of *in vitro* susceptibilities of *M. hyopneumoniae* field isolates found significantly increased MIC values against several antimicrobials [15], aligning with our study’s results. Comparing the inhibition of protein synthesis on the ribosome by tildipirosin and other veterinary macrolides provided insights into the molecular interactions that could explain phenotypic resistance [36]. However, it is crucial to note that some studies have documented high MIC values in *Mycoplasma* species without the presence of genetic markers typically associated with resistance. For instance, in a study on *Mycoplasma gallisepticum*, resistance to tylosin was linked to proteomic changes rather than genetic mutations. This study identified enzymatic activities associated with resistance, such as the overexpression of elongation factors Tu and G, ATP synthase subunit beta, and DnaK-HSP70, suggesting that resistance mechanisms may involve regulatory changes at the proteomic level rather than specific genetic alterations [32]. This is further confirmed by the study of nonpathogenic *Mycoplasma* species isolated from South African poultry showing high MIC values in a wide range of antimicrobials, including macrolides and fluoroquinolones, without genetic markers. These findings suggest that resistance mechanisms are very complex in nature and may be due to efflux pumps, biofilm formation, or metabolic bypass pathways [37,38]. The work of Kobayashi et al. [14] and Stakenborg et al. [9] further supports the idea that elevated MICs can be multifactorial. Both studies reported variable resistance levels to macrolides in *M. hyopneumoniae* and *M. hyorhinis*, with mutations in the 23S rRNA gene contributing to some cases but not all [9,14].

The low genetic diversity observed in the Australian *M. hyopneumoniae* isolates suggests a clonal population structure, likely driven by geographical isolation and limited gene flow. This finding is consistent with the work of Felde et al. [8], who also reported low diversity among isolates from geographically isolated populations. The phylogenetic analysis in this study, showing genetic distinction from isolates in Europe and Asia, supports the notion of regional variation in *M. hyopneumoniae* populations.

A similar study from Australia by Hasoon et al. [39], discussed the clonal expansion of *Mycoplasma bovis* in this ocean-surrounded country. The high level of biosecurity and geographic isolation significantly limits gene flow between herds, accounting for the low genetic diversity observed within the *Mycoplasma* species in Australia. This contrasts with studies in Europe, such as that by Vranckx and colleagues, which show higher genetic diversity among European *M. hyopneumoniae* isolates [7]. The greater diversity in Europe is likely due to a combination of factors, including the continent’s numerous land borders, high-density pig populations, varying levels of biosecurity, and differing management policies across regions. These factors contribute to more frequent gene flow between herds and regions, leading to a more genetically heterogeneous population. Notably, *M. hyopneumoniae* isolates from regions with dense pig populations show significant genetic variation, contrasting with the relatively homogenous population found in Australia. In this respect, the grouping of Australian isolates into a single clade, divergent from other countries, underscores the impact of geographic isolation on the evolution of these strains. The limited diversity in Australia may also influence the development of resistance in the pathogen population, as reduced genetic variation can limit the emergence of resistant strains [11,40] (Vicca et al. 2003; Marois et al. 2007). However, our *in silico* analysis predicts the potential for phenicol resistance in some isolates. Although reports from different parts of the world show relatively low MIC values against phenicols [5,22], the observation that approximately half of our isolates exhibited MIC values that were one-fold higher than the reference strain may indicate a trend towards a decreased phenicol susceptibility of *M. hyopneumoniae* in Australia. This finding highlights the need for continued surveillance to monitor the evolution of resistance in the future. Geographical isolation and strict biosecurity probably enhance the restricted genetic diversity among Australian *M. hyopneumoniae* isolates by limiting gene flow between populations. The selection of only those isolates that were capable of successful *in vitro* culture might have partly influenced the low diversity. This selection bias underlines the need for other methods in order to understand the full genetic pattern of *M. hyopneumoniae* populations, which may be non-cultivable using the traditional techniques today. Future studies will be required to develop novel culture techniques and apply direct genomic approaches, such as metagenomics, in order to get a comprehensive genetic view of this pathogen.

## Figures and Tables

**Table 1:** Primer sequences, product size, and annealing temperatures for molecular detection.

Target Gene	Forward Primer Sequence (5′-3′)	Reverse Primer Sequence (5′-3′)	Product Size (bp)	Annealing Temperature (°C)
IGS	IGS-F GTGGGGATGGATCACCTCCT	IGS-R GCATCCACCAAAAACTCTT	540 (Mhyo) 314 (MhyoR) 450 (Mflo)	55
16S rRNA	16S-F1 GAGCCTTCAAGCTTCACCAAGA	16S-R1 TGTGTTAGTGACTTTTGCCACC	645	56

IGS: 16S–23S rRNA intergenic spacer region; Mhyo: *Mycoplasma hyopneumoniae*; MhyoR: *Mycoplasma hyorhinis*; Mflo: *Mycoplasma flocculare*.

**Table 2:** Primer sequences, product size, and annealing temperatures for MAMA PCR.

Mutated Nucleotide or Amino Acid	Primer Sequence (5′-3′)	Annealing	Amplicon Size (bp)	Target Gene	Antimicrobial Family
23S-F:	AAGGCGCAATGATCTCCCTA	56	171	23S rRNA	Macrolides
23S-R:	CCTTGGATCGACATCTCCCA	56		23S rRNA	Macrolides
23S-2057-F:	GGTTACCCGCATCAAGACTA	56	104	23S rRNA	Macrolides
23S-2058-F:	GTTACCCGCATCAAGACGAG	56	103	23S rRNA	Macrolides
23S-2059-F:	TTACCCGCATCAAGACGGGA	56	102	23S rRNA	Macrolides and Lincosamides
Gyr-F:	CCATCAATTGATCCAAAATT	56	118	DNA Gyrase A	Fluoroquinolones
GyrAla-Rs:	GAAAATACCATCCTCACGG	56	118	DNA Gyrase A	Fluoroquinolones
GyrAla-R:	GGGCGAAAATACCATCCTCAAGC	56	118	DNA Gyrase A	Fluoroquinolones
GyrVal-Rs:	ACCATCGATTCATAGACAGCAG	59	118	DNA Gyrase A	Fluoroquinolones
GyrVal-R:	GGGGCACCATCGATTCATAGACAGCAA	59	118	DNA Gyrase A	Fluoroquinolones
GyrGlu-Rs:	CATTCGCACCATCGCTT	56	118	DNA Gyrase A	Fluoroquinolones
GyrGlu-R:	GGGGCCATTCGCACCATCGTTC	56	118	DNA Gyrase A	Fluoroquinolones
Par-Ser-F:	AATCTGCTAGAGTTGTCGGTG	55	75	Topoisomerase IV	Fluoroquinolones
Par-Ser-R:	CAAGAGCATCATAGATTGGAG	55		Topoisomerase IV	Fluoroquinolones
Par-Ser-R:	CAAGAGCATCATAGATTGCAT	55	87	Topoisomerase IV	Fluoroquinolones
Par-Asp-F:	AATCTGCTAGAGTTGTCGGTG	60	85	Topoisomerase IV	Fluoroquinolones
Par-Asp-R:	GCAAGTCTGACAAGAGCGTC	60		Topoisomerase IV	Fluoroquinolones
Par-Asp-R:	GGGGCGCAAGTCTGACAAGAGCCTT	60	90	Topoisomerase IV	Fluoroquinolones

**Table 3:** MIC distribution table, with MIC50 and MIC90 obtained for 21 *Mycoplasma hyopneumoniae* isolates isolated from Australian pigs.

Antimicrobial	ATCC (µg/mL)	Antimicrobial Concentration (µg/mL)											
		0.125	0.25	0.5	1	2	4	8	16	32	64	MIC50 (µg/mL)	MIC90 (µg/mL)
Aminocyclitol (Spectinomycin)	2	0	0	0	0	48%	38%	14%	0	0	0	2	4
Fluoroquinolons (Enrofloxacin)	0.25	0	4%	48%	48%	0	0	0	0	0	0	1	1
Lincosamides (Lincomycin)	2	0	0	0	0	62%	38%	0	0	0	0	2	4
Macrolides: -Erythromycin	32	0	0	0	0	0	0	0	0	48%	52%	64	64
-Gamithromycin	8	0	0	0	0	0	0	0	0	70%	30%	32	32
-Tildipirosin	2	0	0	0	0	57%	43%	0	0	0	0	2	4
-Tilmicosin	8	0	0	0	0	0	0	9%	72%	19%	0	16	32
-Tulathromycin	2	0	0	0	0	43%	43%	14%	0	0	0	2	4
-Tylosin	0.5	0	0	0	52%	48%	0	0	0	0	0	1	1
Phenicol (Florfenicol)	4	0	0	0	0	0	52%	48%	0	0	0	4	8
Pleuromutilins (Tiamulin)	0.25	0	0	0	4%	34%	62%	0	0	0	0	4	4
Tetracyclines (Tetracycline)	0.25	14%	62%	24%	0	0	0	0	0	0	0	0.25	0.5

**Table 4:** Comparison of antimicrobial MIC_50_ values for ATCC *Mycoplasma hyopneumoniae* isolate with values * reported by Stakenborg et al. [9]. NA: data not available.

Antimicrobial	ATCC MIC50 (µg/mL)	ATCC MIC Values Range; Reported by Felde et al. [8] (µg/mL)
Aminocyclitol (Spectinomycin)	2	1–4
Fluoroquinolones (Enrofloxacin)	0.25	0.04–0.08
Lincosamides (Lincomycin)	2	0.25–1
Macrolides: -Erythromycin	32	8–32 *
-Gamithromycin	8	1–4
-Tildipirosin	2	NA
-Tilmicosin	8	2–8
-Tulathromycin	2	1–4
-Tylosin	0.5	0.25–0.5
Phenicol (Florfenicol)	4	1–2
Pleuromutilins (Tiamulin)	0.25	0.04–0.16
Tetracyclines (Tetracycline)	0.25	0.25–4

## Data Availability

The raw WGS data of the field isolates are available in the SRA under BioProject PRJNA1117611.

## References

[1] Zimmerman J.J., Karriker L.A., Ramirez A., Schwartz K.J., Stevenson G.W. (2019). Diseases of Swine.

[2] López Rodriguez A., Berge A.C., Ramage C., Saltzman R., Domangue R.J., Gnozzio M.J., Benchaoui H.A. (2020). Evaluation of the clinical efficacy of a water soluble formulation of tylvalosin in the control of enzootic pneumonia associated with *Mycoplasma hyopneumoniae* and *Pasteurella multocida* in pigs. Porcine Health Manag..

[3] Maes D., Segales J., Meyns T., Sibila M., Pieters M., Haesebrouck F. (2008). Control of *Mycoplasma hyopneumoniae* infections in pigs. Vet. Microbiol..

[4] Maes D., Sibila M., Kuhnert P., Segalés J., Haesebrouck F., Pieters M. (2018). Update on *Mycoplasma hyopneumoniae* infections in pigs: Knowledge gaps for improved disease control. Transbound. Emerg. Dis..

[5] Thongkamkoon P., Narongsak W., Kobayashi H., Pathanasophon P., Kishima M., Yamamoto K. (2013). *In vitro* susceptibility of *Mycoplasma hyopneumoniae* field isolates and occurrence of fluoroquinolone, macrolides and lincomycin resistance. J. Vet. Med. Sci..

[6] Vicca J., Maes D., Stakenborg T., Butaye P., Minion F., Peeters J., Haesebrouck F. (2007). Resistance mechanism against fluoroquinolones in *Mycoplasma hyopneumoniae* field isolates. Microbial. Drug Resist..

[7] Vranckx K., Maes D., Sacristán R.D.P., Pasmans F., Haesebrouck F. (2012). A longitudinal study of the diversity and dynamics of *Mycoplasma hyopneumoniae* infections in pig herds. Vet. Microbiol..

[8] Felde O., Kreizinger Z., Sulyok K.M., Hrivnák V., Kiss K., Jerzsele Á., Bányai K. (2018). Antibiotic susceptibility testing of *Mycoplasma hyopneumoniae* field isolates from Central Europe for fifteen antibiotics by microbroth dilution method. PLoS ONE.

[9] Stakenborg T., Vicca J., Butaye P., Maes D., Minion F.C., Peeters J., De Kruif A., Haesebrouck F. (2005). Characterization of *in vivo* acquired resistance of *Mycoplasma hyopneumoniae* to macrolides and lincosamides. Microb. Drug Resist..

[10] Tavío M.M., Poveda C., Assunção P., Ramírez A.S., Poveda J.B. (2014). *In vitro* activity of tylvalosin against Spanish field strains of *Mycoplasma hyopneumoniae*. Vet. Rec..

[11] Vicca J., Stakenborg T., Maes D., Butaye P., Peeters J., de Kruif A., Haesebrouck F. (2003). Evaluation of virulence of *Mycoplasma hyopneumoniae* field isolates. Vet. Microbiol..

[12] Wyns H., Meyer E., Plessers E., Watteyn A., De Baere S., De Backer P., Croubels S. (2014). Pharmacokinetics of gamithromycin after intravenous and subcutaneous administration in pigs. Res. Vet. Sci..

[13] Sulyok K.M., Kreizinger Z., Wehmann E., Lysnyansky I., Bányai K., Marton S. (2017). Mutations associated with decreased susceptibility to seven antimicrobial families in field and laboratory-derived *Mycoplasma bovis* strains. Antimicrob. Agents Chemother..

[14] Kobayashi H., Nakajima H., Shimizu Y., Eguchi M., Hata E., Yamamoto K. (2005). Macrolides and Lincomycin susceptibility of *Mycoplasma hyorhinis* and variable mutation of domain II and V in 23S ribosomal RNA. J. Vet. Med. Sci..

[15] Vicca J., Stakenborg T., Maes D., Butaye P., Peeters J., de Kruif A. (2004). *In vitro* susceptibilities of *Mycoplasma hyopneumoniae* field isolates. Antimicrob. Agents Chemother..

[16] Gautier-Bouchardon A. (2018). Antimicrobial Resistance in *Mycoplasma* spp. Microbiol. Spectr..

[17] van der Schalk T.E., Braam J.F., Kusters J.G. (2020). Molecular Basis of Antibiotic Resistance in *Mycoplasma genitalium*. Int. J. Antimicrob. Agents.

[18] Nathues H., Beilage E.G., Kreienbrock L., Rosengarten R., Spergser J. (2011). RAPD and VNTR analyses demonstrate genotypic heterogeneity of *Mycoplasma hyopneumoniae* isolates from pigs housed in a region with high pig density. Vet. Microbiol..

[19] Cook B.S., Beddow J.G., Manso-Silvan L., Maglennon G.A., Rycroft A.N. (2016). Selective medium for culture of *Mycoplasma hyopneumoniae*. Vet. Microbiol..

[20] Hannan P. (2000). Guidelines and recommendations for antimicrobial minimum inhibitory concentration (MIC) testing against veterinary mycoplasma species. Vet. Res..

[21] (2018). Clinical and Laboratory Standards Institute (CLSI). Performance Standards for Antimicrobial Susceptibility Testing.

[22] Felde O., Kreizinger Z., Sulyok K.M., Wehmann E., Gyuranecz M. (2020). Development of molecular biological tools for the rapid determination of antibiotic susceptibility of *Mycoplasma hyopneumoniae* isolates. Vet. Microbiol..

[23] Bolger A.M., Lohse M., Usadel B. (2014). Trimmomatic: A flexible trimmer for Illumina sequence data. Bioinformatics.

[24] Andrews S. (2010). FastQC: A Quality Control Tool for High Throughput Sequence Data. http://www.bioinformatics.babraham.ac.uk/projects/fastqc/.

[25] Bankevich A., Nurk S., Antipov D., Gurevich A.A., Dvorkin M., Kulikov A.S., Lesin V.M., Nikolenko S.I., Pham S., Prjibelski A.D. (2012). SPAdes: A new genome assembly algorithm and its applications to single-cell sequencing. J. Comput. Biol..

[26] Stamatakis A. (2014). RAxML version 8: A tool for phylogenetic analysis and post-analysis of large phylogenies. Bioinformatics.

[27] Didelot X., Wilson D.J. (2015). ClonalFrameML: Efficient inference of recombination in whole bacterial genomes. PLoS Comput. Biol..

[28] Antunes N.T., Tavío M., Assunção P., Rosales R., Aquili V., de la Fe C. (2007). In vitro susceptibilities of field isolates of *Mycoplasma mycoides* subsp. mycoides large colony type to 15 antimicrobials. Vet. Microbiol..

[29] Lysnyansky I., Ayling R.D. (2016). *Mycoplasma bovis*: Mechanisms of resistance and trends in antimicrobial susceptibility. Front. Microbiol..

[30] Pyörälä S., Baptiste K.E., Catry B., van Duijkeren E., Greko C., Moreno M.A. (2014). Macrolides and lincosamides in cattle and pigs: Use and development of antimicrobial resistance. Vet. J..

[31] Kidsley A.K., Abraham S., Bell J.M., O’Dea M., Laird T.J., Jordan D., Mitchell P., McDevitt C.A., Trott D.J. (2018). Antimicrobial susceptibility of *Escherichia coli* and *Salmonella* spp. isolates from healthy pigs in Australia: Results of a pilot national survey. Front. Microbiol..

[32] Xia X., Wu C., Cui Y., Kang M., Li X., Ding S., Shen J. (2015). Proteomic analysis of tylosin-resistant *Mycoplasma gallisepticum* reveals enzymatic activities associated with resistance. Sci. Rep..

[33] Le Carrou J., Laurentie M., Kobisch M., Gautier-Bouchardon A.V. (2006). Persistence of *Mycoplasma hyopneumoniae* in experimentally infected pigs after marbofloxacin treatment and detection of mutations in the parC gene. Antimicrob. Agents Chemother..

[34] Pallarés F.J., Lasa C., Roozen M., Ramis G. (2015). Use of tylvalosin in the control of porcine enzootic pneumonia. Vet. Rec. Open.

[35] Klein U., de Jong A., Moyaert H., El Garch F., Leon R., Richard-Mazet A., Simjee S. (2017). Antimicrobial susceptibility monitoring of *Mycoplasma hyopneumoniae* and Mycoplasma bovis isolated in Europe. Vet. Microbiol..

[36] Andersen N.M., Poehlsgaard J., Warrass R., Douthwaite S. (2012). Inhibition of protein synthesis on the ribosome by tildipirosin compared with other veterinary macrolides. Antimicrob. Agents Chemother..

[37] Beylefeld A., Wambulawaye P., Bwala D.G., Gouws J.J., Lukhele O.M., Wandrag D.B.R., Abolnik C. (2018). Evidence for multidrug resistance in nonpathogenic *Mycoplasma* species isolated from South African poultry. Appl. Environ. Microbiol..

[38] Chernova O., Medvedeva E., Mouzykantov A., Baranova N., Chernov V. (2016). *Mycoplasmas* and Their Antibiotic Resistance: The Problems and Prospects in Controlling Infections. J. Clin. Microbiol..

[39] Hasoon M.F., Jarocki V.M., Mohammed M.H., Djordjevic S.P., Yip H.Y.E., Carr M., Trott D.J. (2023). Antimicrobial susceptibility and molecular characteristics of *Mycoplasma bovis* isolated from cases of bovine respiratory disease in Australian feedlot cattle. Vet. Microbiol..

[40] Marois C., Le Carrou J., Kobisch M., Gautier-Bouchardon A.V. (2007). Isolation of *Mycoplasma hyopneumoniae* from different sampling sites in experimentally infected and contact SPF piglets. Vet. Microbiol..

